# Hepatitis B Virus and DNA Damage Response: Interactions and Consequences for the Infection

**DOI:** 10.3390/v9100304

**Published:** 2017-10-19

**Authors:** Andoni Gómez-Moreno, Urtzi Garaigorta

**Affiliations:** 1 Department of Molecular and Cellular Biology, Centro Nacional de Biotecnología—Consejo Superior de Investigaciones Científicas (CNB-CSIC), Darwin 3, 28049 Madrid, Spain; agomez@cnb.csic.es; 2 Centro de Investigación Biomédica en Red (CIBER) de Enfermedades Hepáticas y Digestivas (CIBERehd), 28029 Madrid, Spain

**Keywords:** hepatitis B virus, DNA damage response, cccDNA, host factors, hepatocellular carcinoma, DNA integration

## Abstract

Hepatitis B virus (HBV) is a major etiologic agent of acute and chronic hepatitis, and end-stage liver disease. Establishment of HBV infection, progression to persistency and pathogenesis are determined by viral and cellular factors, some of which remain still undefined. Key steps of HBV life cycle e.g., transformation of genomic viral DNA into transcriptionally active episomal DNA (cccDNA) or transcription of viral mRNAs from cccDNA, take place in the nucleus of infected cells and strongly depend on enzymatic activities provided by cellular proteins. In this regard, DNA damage response (DDR) pathways and some DDR proteins are being recognized as important factors regulating the infection. On one hand, HBV highjacks specific DDR proteins to successfully complete some of the steps of its life cycle. On the other hand, HBV subverts DDR pathways to presumably create a cellular environment that favours its replication. Direct consequences of these interactions are: HBV DNA integration into host chromosomal DNA, and accumulation of mutations in host chromosomal DNA that could eventually trigger carcinogenic processes, which would explain in part the incidence of hepatocellular carcinoma in chronically infected patients. Unravelling the interactions that HBV establishes with DDR pathways might help identify new molecular targets for therapeutic intervention.

## 1. Introduction

Hepatitis B virus (HBV) represents an important human pathogen causing both acute and chronic hepatitis [1]. It is estimated that over 250 million people are chronically infected and more than 780,000 people die every year due to complications of HBV, including liver cirrhosis and hepatocellular carcinoma (HCC), accounting for more than 50% of all HCC cases worldwide [2,3]. Integrated forms of HBV DNA are found in most of HBV-related HCC cases but they do not contribute to productive infection (reviewed in [4]). A hallmark of HBV persistence is the presence of a long-lasting episomal DNA, known as covalently closed circular DNA (cccDNA), in the nucleus of infected hepatocytes. In order to establish a stable cccDNA pool, HBV hijacks cellular factors that provide, at least in part, the enzymatic activities required for its formation, maintenance, and expression (reviewed in [5]). Most of these factors are involved in DNA damage recognition and repair, and they belong to cellular pathways collectively known as DNA Damage Response (DDR) pathways. As it happens in other virus infections, HBV interacts with some DDR pathways and their components in a reciprocal manner: on one hand, HBV infection deregulates DDR pathways; on the other hand, DDR pathway deregulation interferes with HBV infection. In this review we will discuss the current knowledge on the interactions that HBV establishes with DDR pathways during the infection. We will focus specifically on: (i) the DDR factors involved in cccDNA formation, transcription and degradation, (ii) the HBV factors that modulate DDR pathways, and (iii) the consequences of DDR deregulation for HBV infection.

## 2. Hepatitis B Virus Life Cycle

HBV is a small, enveloped, non-cytolytic virus belonging to *Hepadnaviridae* family [6]. It replicates in quiescent hepatocytes [7] by reverse transcription of a terminally redundant RNA, known as pregenomic RNA (pgRNA) [8]. The infection starts with a low affinity interaction of HBV virions with heparan sulfate proteoglycans present in the cell surface of the hepatocytes [9]. This initial attachment step is followed by a higher affinity binding of the PreS1 domain of the large envelope protein of HBV to its cellular receptor, the sodium taurocholate cotransporting polypeptide (NTCP) [10]. This interaction triggers the internalization of HBV through a clathrin-dependent endocytosis-mediated process [11] that finalizes with the release of the HBV genome-containing capsid into the cytoplasm. The capsid is then imported to the nuclear basket within the nuclear pore [12] from where the HBV genome (known as relaxed circular DNA (rcDNA)) is released into the nucleus. The molecular mechanisms responsible of capsid breakdown and rcDNA release are still poorly understood. rcDNA is then repaired by host enzymes to form cccDNA, which is assembled into a minichromosome [13] and serves as the transcriptional template for the production of all viral messenger RNAs (mRNAs). The cellular RNA polymerase II is responsible for the production of viral transcripts (six unspliced and one spliced). All viral transcripts contain a 5′-capped structure and share a common 3′-end polyadenylation site. These features make them structurally indistinguishable from cellular mRNAs. Transcription is driven by four viral promoters (preS1, preS2, core, and X) and two enhancers (Enh1 and Enh2) distributed throughout the viral genome. Unspliced transcripts encode total of seven viral proteins: the viral polymerase (pol) and core (HBcAg) proteins are produced from the pgRNA; the pre-core protein (e antigen or HBeAg) from the precore mRNA; the large (L), medium (M), and small (S) envelope surface proteins from the PreS1, PreS2, and M/S mRNAs, respectively; and HBx protein from the X mRNA. The main functions of these proteins in the infection have been elucidated over the years (reviewed in [6]). Instead, the function and biological significance of hepatitis B virus spliced protein (HBSP) encoded by the spliced transcript is still uncertain [14,15]. Besides its coding capacity, the pgRNA serves also as template for virus genome replication in a process that is dependent on: (i) the recognition and binding of viral polymerase to the stem-loop structure epsilon (ε) located on the 5′ redundant region of the pgRNA, and (ii) the enzymatic activities of viral polymerase. The specific interaction of viral polymerase and ε in the cytoplasm triggers packaging of the pgRNA inside newly formed capsids and the initiation of the replication process [16]. pgRNA-bound viral polymerase primes the reverse transcription step in which the first DNA strand (minus strand) is generated along with the almost complete degradation of the pgRNA by the RNase H activity of the polymerase. Subsequently, the incomplete second DNA strand (plus strand) is produced through the DNA-dependent DNA polymerization activity of the viral polymerase, generating the partially double-stranded DNA form or rcDNA. This complex replication process produces not only the desired rcDNA molecules but also double-stranded linear DNA (dslDNA) forms that account for up to 10% of all viral DNA molecules [17]. Newly formed replication-derived capsids can be transported back to the nucleus, thereby amplifying the cccDNA pool in what is known as the intracellular recycling pathway [18]. On the other hand, infectious viral particles (known as Dane particles) are produced upon envelopment of replication-derived capsids and they exit the cells via the secretory pathway or via a different pathway that depends on the formation of multivesicular bodies [19]. Infected hepatocytes also release huge amounts of HBeAg and hepatitis B surface antigen (HBsAg) subviral particles. HBeAg and HBsAg have been proposed to function as immune modulators during HBV infection [20,21].

## 3. Brief Overview of DNA Damage Response Pathways

DNA damage response (DDR) pathways orchestrate the recognition and repair of DNA lesions in the genome that occur during normal cellular processes or are introduced by exogenous agents. Many viruses interact with DDR pathways in such ways that they deploy strategies designed to either activate or inhibit specific DDR pathways or their components to ensure successful completion of their replication cycles [22]. More than 200 proteins have been ascribed to DDR pathways in mammalian cells. DDR proteins are classified according to their specific function as sensors, transducers, mediators, and effectors [23]. The type of DNA lesion dictates the specific DDR pathway(s) involved in the repair. The most common DNA lesions include: nitrogenous base loss or modifications, single strand breaks (SSB), double strand breaks (DSB), and covalent crosslinking of two DNA strands, among others [24]. Upon DNA damage, phosphatidylinositol 3-like-kinases i.e., Ataxia telangiectasia-mutated (ATM), Ataxia telangiectasia-mutated and Rad3-related (ATR), and DNA-dependent protein kinase (DNA-PK) are activated. This activation is followed by subsequent phosphorylation of mediators and effectors which in turn initiate DNA repair [25,26,27]. ATR is usually activated when DNA lesion results in SSBs [26], while ATM- and DNA-PK-dependent pathways are triggered by DSBs [25,27]. Strand break repair is typically carried out by one or more of the following pathways: SSB repair pathways that comprise the long and short pathways [28]; and DSB repair pathways that include the non-homologous end joining (NHEJ), both the classical (cNHEJ) and the microhomology-mediated end joining (MMEJ), and the homologous repair (HR) pathway [29]. Base excision repair (BER) [30], nucleotide excision repair (NER), including global genome and transcription-coupled repair (GG-NER and TC-NER) [31], and mismatch repair (MMR) [32] pathways are responsible of the repair of missing or modified nitrogenous bases. Fanconi Anemia (FA) pathway is responsible for the repair of covalent DNA interstrand cross-links [33]. It should be noted that the activity of these pathways is in many cases tightly coupled to the cell cycle. For instance, HR activity is high during DNA replication in S phase [29]. Under certain circumstances when DNA lesions accumulate and overcome the cellular repair capacity, cell cycle arrest, senescence, or apoptosis might occur [34,35].

## 4. DDR Factors and Pathways Involved in cccDNA Formation, Expression and Degradation

### 4.1. Formation of cccDNA

**Figure 1:**
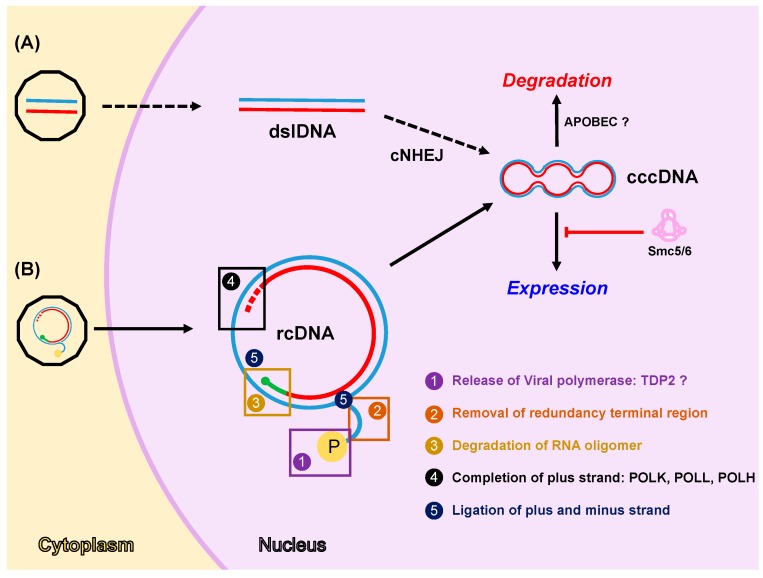
DNA damage repair (DDR) factors regulate cccDNA formation, expression, and degradation. Two pathways for cccDNA formation have been proposed: the first pathway (**A**) is based on the transformation of dslDNA derived from DHBV replication into cccDNA by the classical non-homologous end joining pathway (cNHEJ); the second pathway (**B**) is based on the transformation of human HBV rcDNA, derived from incoming virions or intracellular recycling pathway, into cccDNA and involves several enzymatic activities. (1) Viral polymerase release from minus strand might be mediated by cellular TDP2 protein. (2 and 3) Removal of redundancy terminal region and degradation of RNA oligomer could be mediated by cellular exo- and/or endonucleases. (4) Completion of the plus strand involves the activity of cellular DNA polymerases i.e., POLκ, POLλ, and POLη. (5) Ligation of plus and minus strands is carried out by cellular ligases. Once formed, cccDNA is assembled into a minichromosome and it functions as the transcriptional template to generate all viral mRNAs. Cellular factors can regulate cccDNA homeostasis by silencing its expression (i.e., Smc5/6) or inducing its degradation (i.e., APOBEC proteins). Note that: minus and plus strand DNAs are shown in blue and red, respectively; capped RNA oligomer is depicted in green; and viral polymerase (P) is represented as a yellow circle in the rcDNA. Question marks indicate conflicting results published in the literature. Dashed arrows shown in the first pathway (**A**) indicated that there is no evidence that this pathway occurs in human HBV cccDNA formation.

The HBV genome (rcDNA) is a partially double-stranded DNA molecule of around 3.2 kb in length with distinct structural features reminiscent of its complex DNA replication cycle [6]. The minus strand DNA is complete and more than genome length in size. It contains the viral polymerase covalently bound through a tyrosine residue to the 5′ terminal redundancy region. Instead, the plus strand DNA is incomplete at its 3′-end and contains an RNA oligomer at its 5′-end, which derives from the reverse transcription. Neither the plus strand nor the minus strand is covalently closed. Thus, transformation from rcDNA to cccDNA encompasses a set of processes that include: release of viral polymerase and removal of redundancy terminal region from the minus strand, degradation of RNA oligomer from the plus strand, completion of the plus strand and ligation of both strands (Figure 1). It is important to mention that the spatial and temporal distribution of these processes, which lead to the production of cccDNA from rcDNA, is still uncertain and theoretically they could happen either sequentially or in some cases even simultaneously. The nature and specific contributions of viral and cellular factors to these processes are still largely unknown in part because of the lack of suitable experimental systems in the past. However, the discovery of NTCP as the HBV receptor and the development of hepatic cell lines susceptible to HBV infection e.g., the HepG2-NTCP [10], have provided an easily accessible platform for studying early steps of HBV infection including the transformation of the incoming rcDNA into cccDNA.

The presence of viral polymerase attached to the minus strand in the rcDNA molecule opened the possibility that viral polymerase itself is responsible for the completion of the plus strand DNA during cccDNA formation. However, infection experiments performed in the presence of highly specific and potent viral polymerase inhibitors have demonstrated that viral polymerase activity is not necessary for cccDNA accumulation during de novo HBV infection [36]. Since the polymerase is the only viral protein with enzymatic activities, these results strongly suggest that cccDNA formation depends primarily on cellular factors. In fact, in the last years, cellular proteins, some of which are involved in DDR pathways, have been identified as factors required for cccDNA formation [36,37,38].

A key step in cccDNA formation is the release of viral polymerase from the minus strand. Tyrosil DNA phosphodiesterase 2 (TDP2) was the first cellular protein identified as the enzyme involved in this step [37]. The authors demonstrated that human and chicken TDP2 cleaved the tyrosil-DNA bond from authentic human and duck hepatitis B virus (DHBV) rcDNA in vitro, and they showed that depletion of TDP2 significantly slowed down the conversion of rcDNA to cccDNA in cell culture. Although other groups have reported similar results in vitro, they were not able to confirm the TDP2 dependency for cccDNA formation in HBV-infected HepG2-NTCP cells [39]. In fact, they reported contradictory results showing that TDP2 could actually inhibit HBV cccDNA formation. These apparent discrepancies could reflect fundamental differences between DHBV and HBV infection as it has been previously shown for the kinetics and mechanisms of the intracellular nucleocapsid recycling between these virus infections [40]. These results may also suggest the existence of other protein(s) and/or pathway(s) involved in the polymerase release from minus strand and they may highlight the existence of functionally redundant pathways that could operate independently of each other and in a virus-specific manner.

Completion of plus strand is another essential process for cccDNA formation. Since viral polymerase enzymatic activity is dispensable for cccDNA formation, one or more cellular polymerases must be responsible for completion of the gap in the plus strand of rcDNA. Genetic siRNA screening and CRISPR/Cas9 genome editing system approaches were used to demonstrate that cellular DNA polymerase κ (POLκ) and, to a lesser extent, DNA polymerases POLη and POLλ play a role in plus strand completion [36]. Whether each of the cellular DNA polymerases has a redundant or distinct role in cccDNA formation remains to be elucidated.

Cleavage or degradation of the RNA oligomer present at the 5′-end of the plus strand and removal of the redundant terminal region of the minus strand are pre-requisites prior to DNA strand ligation and cccDNA formation. The identity of the cellular factors required for these activities is still unknown. However, given the enzymatic activities theoretically involved in these processes e.g., exonuclease and/or endonuclease and ligase activities, it is conceivable that these factors belong to DNA repair pathways that remain active in quiescent hepatocytes since HBV replicates at this cellular stage. Following this argumentation, it has been suggested that cellular endonuclease XPG or exonuclease Exo1 and the complex XRCC1-Lig3 could be involved in RNA oligomer degradation and DNA strand ligation, respectively [36]. The latest is supported by the fact that the XRCC1-Lig3 complex is required for the ligation of NER induced breaks in quiescent cells [41] and POLκ together with POLδ and POLε fill single-stranded DNA gaps in NER pathway [42]. Biochemical and genetic experiments will be needed to elucidate the requirement of these and other cellular factors in the rcDNA to cccDNA transformation.

An alternative pathway for cccDNA formation has been proposed for DHBV and it is based on the direct transformation of dslDNA into cccDNA [43,44]. dslDNA is formed as a consequence of a priming failure event during reverse transcription that leads to the synthesis of dslDNA instead of rcDNA [17]. On one hand, dslDNA is thought to be the predominant precursor for integration in the host genome [45]. On the other hand, as deletions and insertions around dslDNA junctions have been detected in DHBV cccDNA molecules, dslDNA coming either from incoming virions or intracellular recycling pathway has been proposed as DHBV cccDNA precursor [43,44]. Experiments carried out with DHBV in a CHO-derived cell line showed that ku80, a sensory component of the cNHEJ repair pathway, was necessary for cccDNA formation from dslDNA but not from rcDNA [46]. Collectively, these data suggest that the cNHEJ pathway is involved in cccDNA formation from dslDNA, at least in the DHBV system. Further experimentation would be required to test whether this could be extended also to human HBV infection. However, important differences in the kinetics and mechanisms of the intracellular nucleocapsid recycling exist between DHBV and HBV [40] that could be fundamental for the different role of dslDNA in DHBV and HBV cccDNA formation.

### 4.2. Expression from cccDNA

Once cccDNA is formed and assembled into a minichromosome [13] viral transcription occurs. HBx mRNA and protein are expressed at very early stage upon infection [47]. HBx expression has been shown to be necessary for HBV gene expression in all cell culture models reported to date except in the Huh7 cell line system [48,49]. This requirement is linked to HBx transactivation activity, which in turn relies on its ability to bind to DNA damage binding protein 1 (DDB1). DDB1 interacts with culling 4 (cul4) as part of an E3 ubiquitin ligase complex responsible of the polyubiquitination and proteasome-dependent degradation of target proteins [50]. HBV hijacks the DDB1-cul4 protein complex system to mediate the degradation of the structural maintenance of chromosomes complex 5 and 6 (Smc5/6), thereby allowing viral transcription from cccDNA molecules [51,52]. Collectively, these data indicate that Smc5/6 is an HBV restriction factor that is rapidly degraded upon infection through an HBx-dependent mechanism to allow viral gene expression and replication to occur. However, it is not yet clear how Smc5/6-mediated silencing is avoided in order to express HBx from newly synthesized cccDNA early after infection. HBx mRNA has been detected in cell culture-derived virus preparations and in plasma from chronically HBV-infected patients [47]. However, packed HBx mRNA levels are not sufficient to confer escape from Smc5/6-mediated silencing [47]. Thus, an early HBx mRNA transcription could happen even before the Smc5/6 complex had detected and silenced the expression from cccDNA.

The mechanism by which Smc5/6 recognizes cccDNA is still unknown. It appears that the recognition is not sequence specific because the HBx transactivation activity, which is dependent on Smc5/6 degradation, is observed in a variety of cellular and viral promoter and regulatory elements only when they are present in an episomal context but not when the same elements are integrated in the host chromosomal DNA [53]. Recent data suggest that Smc5/6 localization in promyelocytic nuclear bodies (PML-NBs) might be required for cccDNA silencing [47]. Unravelling the molecular mechanisms by which Smc5/6 specifically silences episomal elements might lead to the discovery of new therapeutic approaches to target not only chronic HBV infection but also other episomal virus infections.

### 4.3. Degradation of cccDNA

Non-cytolytic clearance of cccDNA from HBV-infected hepatocytes was first reported by Guidotti and colleagues in acutely infected chimpanzees [54], however, the underlying mechanism remains elusive. In this context, nonhepatotoxic degradation of nuclear HBV cccDNA by cytokine and/or lymphotoxin-β receptor mediated activation of apolipoprotein B mRNA editing enzyme catalytic polypeptide-like 3B (APOBEC3B) was recently reported [55]. The authors proposed that DNA glycosylases could recognize APOBEC3B-mediated deamination of cccDNA and create apurinic/apyrimidinic (AP) sites. These AP sites would be then degraded by cellular endonucleases leading to cccDNA degradation rather than to its repair by BER pathway, as occurs when AP sites are created in chromosomal DNA. In contrast, it has been recently shown that intrahepatic cccDNA and replicative DNA levels do not correlate with APOBEC gene expression in HBV chronically infected patients [56], suggesting that lymphotoxin-β receptor pathway has no mayor impact on cccDNA metabolism in chronic HBV infection. In agreement with this, Seeger and colleagues investigated whether activation of APOBEC3B by interferon α (INFα) and CRISPR/Cas9 endonuclease directed to cccDNA could act synergistically in cccDNA inactivation. They found that Cas9-mediated cleavage of HBV DNA was 15,000 times more efficient that APOBEC-mediated cytosine deamination induced by INFα treatment [57]. Characterization of the complete spectrum of mutations generated by Cas9 cleavage suggested that cccDNA is repaired by cNHEJ pathway and evidence of cccDNA deamination was scarce. Thus, even if APOBEC3B induced deamination on cccDNA, it appeared that deaminations were repaired by the BER pathway. Other proteins belonging to the APOBEC family, such as APOBEC3G [58] or activated induced cytosine deaminase (AID) [59] have been implicated in HBV RNA deamination after pgRNA encapsidation. In summary, the target of APOBEC proteins (RNA or DNA) as well as the biological significance of their activity are controversial at this moment and further experiments are needed to determinate their role during HBV infection, specifically on cccDNA degradation.

## 5. HBV Factors Interfere with DDR Pathways

### 5.1. HBx Protein and DDR

HBx is a pleiotropic viral protein that is expressed at low levels and very early during infection [47]. It plays a central role in HBV gene expression by inducing the degradation of the Smc5/6 complex [51] and it is believed to be an important factor in viral pathogenesis [60]. HBx expression is observed in 20–50% of HCCs [61] and antibodies against HBx have been detected in HBV chronically infected patients [62]. HBx expression in HCC is thought to be derived from HBV DNA integration into host chromosomal DNA [63,64]. Since HBV integration occurs by illegitimate recombination and it is not carried out by viral proteins [43], the intracellular levels of HBx may be different depending on the genomic context where HBx is integrated. Thus, the interactions that HBx establishes with host factors could be highly dependent on the HBx expression levels achieved in different stages of the infection. Taking all of the above into account, overexpression of HBx protein may not be the best way to study its biological properties, especially in the early stages of the infection, before viral DNA integration occurs, and when its expression level is still low. Despite this important consideration, HBx protein overexpression strategies have been widely used to identify and study interactions between HBx and host cellular proteins.

#### 5.1.1. HBx Interaction with DDB1-Cul4 Complex

**Figure 2:**
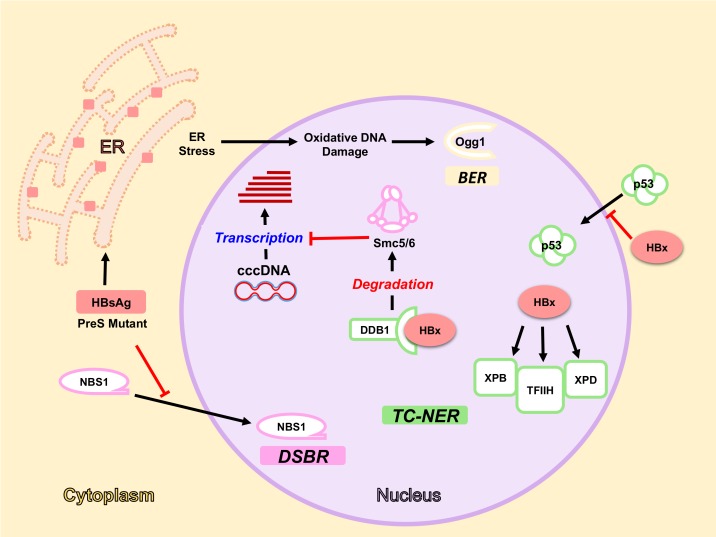
HBV components interact with DDR pathways and proteins. HBx arrests p53 in the cytoplasm and interacts with different factors in the nucleus e.g., DDB1, XPB, XPD, and TFIIH. HBx interaction with TFIIH components XPB and XPD interferes with TC-NER activity. HBx-DDB1 interaction induces Smc5/6 degradation, thereby allowing transcription from cccDNA. Accumulation of wild-type HBsAg and PreS mutant proteins in the ER induces Ogg1 expression through ER Stress and oxidative damage, thereby activating BER pathway. On the other hand, they inhibit NBS1 nuclear translocation affecting DSBR. Proteins of different DDR pathways are represented with different colours: TC-NER in green, DSBR in pink, and BER in yellow. Red bars mean inhibition.

Interaction of HBx with DDB1 protein has been known for long time [65]. This interaction was initially implicated in processes such as: sensitization of liver cells to ultraviolet radiation [66], apoptosis of dividing hepatocytes, S phase progression, chromosome segregation defects, and multi nucleation [67]. More recently, what appears to be its main role during infection was elucidated i.e., induction of Smc5/6 degradation [51] (Figure 2). Since HBx interacts with DDB1 to mediate Smc5/6 degradation and Smc5/6 participates in chromosome maintenance [68], it appears that apoptosis, chromosome segregation, and S phase progression could be all related to Smc5/6 functions.

It was recently shown that depletion of PML and sp100 proteins, components of the PML-NBs, altered the nuclear distribution of Smc6, allowing normal HBV gene expression in the absence of HBx protein [47]. This suggests that the Smc5/6-mediated silencing of cccDNA expression might be dependent on the localization of Smc5/6 in PML-NBs. PML-NBs have also been implicated in DDR, apoptosis, and chromosome segregation [69]. Thus, it would be interesting to go deeper into the underlying mechanisms of these events to understand whether or not they are related to each other.

Binding of HBx protein to Cul4-DDB1 complex could affect the normal physiological functions of the complex, for instance by interfering with the degradation of other cellular target proteins distinct from the Smc5/6 complex. This seems to be the case of the arginine methyltransferase 1 protein (PRMT1), which accumulates to higher levels in HBx expressing cells [70]. The higher PRMT1 accumulation could have undesired consequences for cellular homeostasis, but also for HBV infection. In this regard, PRMT1 was shown to be an HBV restriction factor that binds to cccDNA and suppresses HBV transcription in a methyltransferase activity-dependent manner [71]. Thus, an increased PRMT1 activity could theoretically be detrimental for HBV by inhibiting virus transcription. However, PRMT1 activity is suppressed instead of stimulated in HBV-replicating cells, possibly due to HBx capacity to bind to PRMT1 and inhibit its enzymatic activity [71]. Further experiments will be required to understand the biological significance of the interaction between PRMT1, Cul4-DDB1, and HBx in the context of an HBV infection.

Since HBx functions as a DDB1 Complex Associated Factor (DCAF) and new interactions with other E3 ligases and the ubiquitin proteasome system are now being discovered, the relationship between HBx and these cellular systems could be more complex than previously anticipated. For further reading about this topic we refer to [72].

#### 5.1.2. HBx and NER Pathway

Several evidences support the notion that HBV, through HBx protein, interacts physically and functionally with the NER pathway. It was first reported that HBx interacts with DDB1 protein [65] and that DDB1 plays a role in the repair of UV-induced DNA lesions through NER pathway [73,74]. Thus, Becker and colleagues tested the hypothesis that HBx could interfere with NER activity by binding to DDB1 [75]. They demonstrated that HBx protein expression inhibits the repair of UV-induced DNA lesions. Using a panel of HBx mutant proteins displaying a range of DDB1 interaction capacities, the authors concluded that the ability of HBx protein to inhibit the NER pathway was not absolutely dependent on the HBx–DDB1 interaction, suggesting that HBx could interact with other cellular factors affecting NER activity. In agreement with this, it was later shown that HBx interacts with XPB and XPD helicases, components of the TFIIH complex [76]. This interaction led to defects in the DNA repair of UV-induced lesions, suggesting that HBx protein interferes with the NER pathway, at least in part, by binding physically to some of its components. Moreover, reduction of XPB and XPD gene expression by HBx protein was also reported [77]. TFIIH is known to be regulated by p53, and HBx expression was shown to be sufficient to arrest p53 in the cytoplasm of HepG2 cells [78]. Thus, expression of HBx could affect the NER pathway directly by inhibiting TFIIH component expression and/or function (p53 independent) [76,77] or indirectly through cytoplasmic sequestration of p53 (p53 dependent) [78]. In fact, HBx protein inhibited the TC-NER pathway in both p53-sufficient and p53-deficient human cells [76]. In contrast, HBx did not impair the repair of a defined 1,3-intrastrand d(GpTpG)-cisplatin lesion of a cccDNA substrate, which is not a suitable template for transcription and it is repaired by activation of GG-NER [79]. Thus, the authors concluded that HBx does not interfere with the GG-NER pathway and suggested that HBx would only have inhibitory effects on transcription-coupled events, in agreement with previous observations. It is worth mentioning that the TC-NER rather than the GG-NER pathway is the main active NER pathway in quiescent primary rat hepatocytes [80]. Collectively, the data discussed above suggest that HBV interferes with TC-NER-mediated DNA repair. Since most of the HBx interactions and effects described above were obtained in HBx overexpression systems, it will be interesting to confirm those observations in more physiologically relevant model HBV infection systems. These studies would help elucidate the biological significance of TC-NER pathway deregulation in HBV infection.

### 5.2. HBsAg, ER Stress and Oxidative DNA Damage

The HBsAg is a subviral particle comprised of the three HBV envelope proteins L, M, and S (reviewed in [6]). HBV envelope protein expression is observed in around 30% of HCCs [81] and its expression in HCC is thought to be dependent on integrated forms. One of the hallmarks of HCC is the presence of preneoplastic type II ground glass hepatocytes harbouring the HBV pre-S_2_ mutant large surface protein (LHBS) [82]. Accumulation of wild type and mutant HBV envelope proteins in the ER triggers ER stress, leading to an increased production of reactive oxygen species (ROS) [83]. As a consequence of ROS production and to avoid the accumulation of oxidative lesions in the DNA, upregulation of the 8-oxoguanine glycosylase 1 (ogg1), a recognition factor of oxidative DNA damage, occurs in pre-S_2_ mutant expressing cells [83]. These events trigger activation of BER pathway, a mayor pathway for oxidative DNA damage repair. Interestingly, it has been recently reported that a pre-S_2_ mutant protein blocks the nuclear translocation of the Nijmegen breakage syndrome 1 (NBS1) protein, which is implicated in homologous recombination repair pathway, causing genomic instability and an increased global gene copy number variations [84]. Moreover, ER stress-independent signals produced by pre-S_2_ mutants may enhance DNA damage by stimulating cell cycle progression or upregulating telomerase activity [85,86]. Collectively, it seems that mutations in the pre-S domain of HBsAg, particularly the pre-S_2_ mutants, exacerbate DNA damage by distinct mechanisms including mislocalization of DNA repair proteins (i.e., NBS1) or by promoting ER stress, production of ROS and oxidative DNA damage. However, analysis of oxidative DNA markers in liver biopsy samples from HBV-infected HCC patients did not show any correlation with the presence of HBsAg pre-S mutations [87]. Therefore, further research on the functions and interactions of wild type and mutant envelope proteins will be required to determine the contribution of HBV envelope proteins to DNA damage and hepatocarcinogenesis.

## 6. Consequences of DDR Deregulation for HBV Infection

As discussed above, HBV interferes with DDR pathways and their components in such ways that efficient DNA repair might get compromised. Thus, deregulation of DDR pathways might directly affect HBV infection and HBV-induced pathogenesis. In this regard, many studies have focused on the role of DDR pathways in HBV DNA integration into host chromosomal DNA. It should be noted that the molecular mechanisms and the clinical implications of HBV integration have been recently reviewed [88], therefore, we will focus specifically on the role of DDR pathways in HBV integration, HBV replication, and HBV-induced HCC.

### 6.1. DNA Damage Response and HBV Integration

One of the first direct pieces of evidence that DNA damage promotes HBV de novo integration into cellular chromosomal DNA was reported by Dandri and colleagues [89]. They analysed the effect of H_2_O_2_ and PARP1 inhibitor treatment in new HBV integration events during four generation of subcloning of a human hepatoblastoma-derived cell line stably transfected with HBV DNA (i.e., HepG2.2.15 cell line). They observed that induction of oxidative DNA damage by H_2_O_2_ addition to the medium or inhibition of DNA repair by treatment with a PARP1 inhibitor, led to significantly higher rates of HBV DNA integration. Since oxidative stress induces DSBs and PARP1 is involved in the DSB repair pathway [90], these data suggested that the presence of DSBs in host chromosomal DNA promotes HBV integration. Another conclusion that arose from these studies is that HBV integration is favoured when DSBs induced by exogenous agents are not correctly repaired. These results were later on confirmed in the DHBV system using a brilliant and completely different approach [91]. On one hand, the authors engineered a LMH chicken hepatoma cell line containing a unique I-SceI homing endonuclease integration site and transfected I-SceI expressing vector to induce site-specific DSBs. On the other hand, they transfected a DHBV mutant genome-containing plasmid that is defective in plus strand primer translocation, resulting in a 1:1 ratio of dslDNA to rcDNA. They observed that dslDNA genome was the preferential substrate for integration and that DHBV integrated stably at the specifically engineered DSB site. Collectively, these data suggest that Hepadnaviral DNA integration occurred at sites of DNA damage. Since the approaches described above were based on HBV replication systems, in the absence of the initial steps of virus infection, it would be interesting to confirm that HBV DNA integration occurs preferentially in DNA damage sites also during a de novo HBV infection. To do so, the CRISPR/Cas9 genome-editing system could be used to generate sequence specific DSBs in HBV infection susceptible cells. Nevertheless, it seems that double-strand breaks and possibly other DNA lesions, which repair transitions through a DSB step, could generate integration sites for HBV.

An open question is whether DSBs or other DNA damage lesions, induced by exogenous agents, could happen also in cccDNA molecules, and if so, whether they could trigger the transformation of cccDNA into dslDNA, the preferential substrate for integration. The likelihood of a DNA lesion to occur in a cccDNA molecule would be rather low compared to that in cellular chromosomal DNA given the relatively small size of the cccDNA molecule (~3 × 10^3^ base pairs) compared to the size of cellular chromosomal DNA (~3 × 10^9^ base pairs). Thus, the major contribution of DNA damage to HBV integration seems to be the generation of integration sites in the host chromosomal DNA. In agreement with this, DSBs artificially generated in cccDNA molecules by the CRISPR/Cas9 system were efficiently repaired by the cNHEJ pathway, suggesting that even if cccDNA was damaged it could be repaired by cellular DDR pathways [57]. This raises the question of whether the exact same DDR components and pathways are responsible of the repair of damaged episomal DNA and damaged chromosomal DNA. Addressing this question would help understand the mechanisms involved in cccDNA repair and also in cccDNA formation, from dslDNA and rcDNA. It would also help the design of new DDR-targeting therapeutics for the treatment of episomal DNA virus infections.

The molecular mechanism(s) of HBV integration is(are) still unclear and it could occur through the cNHEJ or MMEJ pathway (reviewed in [88]). High-throughput viral integration detection analysis has recently revealed an enrichment of microhomology sequences between host DNA and integrated HBV DNA, near the site of integration, suggesting that MMEJ may be the mayor pathway involved in HBV integration [92]. The involvement of the MMEJ pathway in human papilloma virus DNA integration in cervical cancer has also been proposed [93]. It would be interesting to take advantage of specific DDR pathway chemical inhibitors and genetic manipulation of DDR components to further study the contribution of the different DDR pathways to HBV DNA integration in HBV infection susceptible cell lines.

### 6.2. DNA Damage Response and HBV Replication

HBV infection is tightly associated with cellular metabolism and the cell cycle. HBV infects quiescent hepatocytes [94] where the intracellular dNTP concentration is particularly low. Reduced dNTP pool availability could theoretically prevent or inhibit HBV replication. However, each HBV-infected hepatocyte produces an average of 50 to 300 virions per day [95]. Therefore, it seems conceivable that in order to boost its replication, HBV has evolved a strategy to increase the intracellular dNTP pool [96]. In fact, HBV infection activates dNTP synthesis by inducing ribonucleotide reductase (RNR) R2 subunit gene expression, a rate-limiting enzyme for dNTP synthesis [96,97]. The R2 subunit gene expression is transcriptionally repressed in quiescent cells by binding of the regulatory factor x1 (RFX1) to the R2 promoter [98]. Two distinct mechanisms have been proposed for the R2 subunit gene induction. The first one is based on the expression of HBx protein that blocks the access of RFX1 to the R2 promoter, thus activating its transcription [96]. The second mechanism is based on HBV-induced DDR activation that involves activation of Chk1, downstream phosphorylation of the transcription factor E2F1, and release of RFX1 repressor from the R2 promoter [97]. These cascades of events trigger the transcriptional upregulation of the R2 subunit enabling enhanced RNR activity and dNTP production that in turn increases HBV DNA replication.

Another consequence of the HBV-induced Chk1-dependent E2F1 activation is the transcriptional upregulation of p73 [99], accumulation of which promotes apoptosis, at least, in rat hepatocytes [100]. Likewise, HBV induction of ATR pathway, via p38MAPKα, stabilizes p53 and induces apoptosis [101]. Despite activation of the above-mentioned pro-apoptotic pathways, the HBV-infected cells survive, suggesting that HBV stimulates mechanisms to counteract those pathways. One of these mechanisms involves activation of Polo-like kinase 1 (Plk1) by HBx protein [102]. On one hand, Plk1 associates, phosphorylates and destabilizes p73, thereby inhibiting its pro-apoptotic activity [103]. On the other hand, Plk1 was recently shown to be a host proviral factor associated with the biogenesis of nucleocapsids by accelerating the proteasomal degradation of two host restriction factors, SUZ12 and ZFN198 [102,104]. Another example of HBV-induced anti-apoptotic mechanism has been already mentioned in this review and involves the cytoplasmic sequestration of p53 by HBx protein [78].

### 6.3. DNA Damage Response and HBV-Induced HCC

It is estimated that 10–15% of human cancers are attributable to viral infections [105] and HBV infection is known to be a major cause of HCC worldwide [2]. Modulation of DDR pathways by viruses have been proposed as a mechanism involved in carcinogenesis since viruses often impede the proper repair of DNA lesions leading to mutations, chromosomal aberrations, and genomic instability, hallmarks of human cancers [22]. As discussed earlier, HBV establishes multiple interactions with DDR proteins and pathways, presumably to create a favourable environment for the infection. Collateral damages of these interactions lead to disease progression and HCC development. Although HBV-induced carcinogenesis has been an active topic of research for many years, the current knowledge of the mechanisms driving HCC is still incomplete.

Pathogenic processes leading to HCC may involve three stages: initiation, promotion, and progression [106]. Initiation is the fixation of mutations in host chromosomal DNA via cell division. Promotion corresponds to clonal expansion of mutated hepatocytes that have an increased risk of oncogenic transformation, while progression includes all the steps by which members of these clonal populations evolve to become cancer cells. Clonal expansion during promotion may presumably involve the emergence of virus-resistant hepatocytes that do not support virus replication due to immune selection [6]. High throughput sequencing analysis of tumour and matched non-tumour liver tissue samples from HBV-induced HCCs has revealed high sequence heterogeneity among different tumour samples, making it very difficult to identify key genetic events responsible for the progression phase [92].

Several mechanisms have been proposed to explain the carcinogenic potential of HBV. On one hand, inflammation, oxidative stress, and inadequate humoral and cellular immune responses associated to chronic HBV infection have been identified as important drivers of HCC. Chronic immune-mediated liver cell injury triggers HCC development in the absence of other carcinogenic stimuli [107]. On the other hand, direct effect of viral factors (i.e., HBx and HBsAg) in the induction of DNA damage or impairment of DDR pathways have been proposed as alternative mechanisms for HCC induction [108]. In this regard, many of the HBV–DDR interactions summarized in this review could potentially lead to the generation of carcinogenic mutations. Moreover, increased DNA damage enhances HBV DNA integration in host chromosomal DNA [89,91]. In fact, HBV DNA integration events occur at low frequency (0.01–0.1% of infected hepatocytes) in transient infections [109]. However, integration events are more frequent in persistent infections and they are present in 80–90% of HBV related HCCs [110], suggesting an association between HBV DNA integration and HBV-induced HCC. Even though integration of HBV DNA occurs in random sites within host chromosomal DNA [111], recurrent integration sites in or near specific genes have been reported in 10–15% of HCCs [110,112]. Moreover HBV integration has been associated in some cases with chromosomal instability, chromosomal rearrangements, and copy number variations [92,110], all of which could contribute to HCC development.

In summary, it seems clear that HBV-induced carcinogenesis is a multifactorial process in which several mechanisms (e.g., insertional mutagenesis, chromosomal instability) may operate individually, simultaneously, or sequentially to promote HCC development. Thus, further research will be required to elucidate the complex interaction pattern and the contribution of each factor to each step during HCC development.

## 7. Final Remarks

Interactions between viruses and cellular DNA damage response pathways are being recognized as important determinants regulating not only the outcome of virus infections, but also the pathogenesis associated with those infections. HBV infection is an example of the different types of interactions that viruses might establish to productively infect the host and produce disease. On one hand, HBV hijacks cellular factors, including some DDR proteins, to successfully complete some of the steps of its life cycle. This is a very active area of research at this moment and it is expected to lead to the discovery of new DDR factors that regulate the infection. On the other hand, HBV proteins have the potential to deregulate specific DDR pathways directly by binding to DDR proteins or indirectly by manipulating intracellular signalling pathways that affect DNA repair. In this regard, we should keep in mind that many of the virus–host protein interactions and their functional consequences described in the literature have been obtained in viral protein overexpression systems. Thus, the interpretation of the results and the conclusions derived from them should be carefully done. The recent development of new HBV infection cell culture systems (e.g., HepG2-NTCP) and animal models (e.g., humanized mouse models) will provide the possibility to confirm or refute some of the interactions described in other less physiologically relevant systems.

Currently approved therapies (e.g., nucleoside analogues) for the treatment of chronic HBV infection are very effective in suppressing virus replication and viremia but they are not curative because they fail to completely eliminate the cccDNA pool, a hallmark of persistency [113,114,115]. Therefore, there is a real need for the development of new antiviral drugs that will eliminate or permanently silence cccDNA. In this regard, a lot of efforts are being made to understand basic aspects of cccDNA biology that could guide the development of such drugs. Given the direct effects of DDR pathways in DNA homeostasis, it is not surprising that research focused on HBV–DDR interactions is currently expanding and it is expected to lead to the discovery of new molecular targets for drug development. Thus, unravelling the complex HBV–DDR interactions regulating the infection may light up the path towards the HBV cure.
